# Success and failure factors of patient recruitment for industry-sponsored clinical trials and the role of the electronic health records—a qualitative interview study in the Nordic countries

**DOI:** 10.1186/s13063-022-06144-9

**Published:** 2022-05-12

**Authors:** Niina Laaksonen, Mia Bengtström, Anna Axelin, Juuso Blomster, Mika Scheinin, Risto Huupponen

**Affiliations:** 1 Institute of Biomedicine, University of Turku, Turku, Finland; 2 Pharma Industry Finland, Helsinki, Finland; 3 Pharmaceutical Sciences Laboratory, Åbo Akademi University, Turku, Finland; 4 Department of Nursing Science, University of Turku, Turku, Finland; 5 Department of Cardiology, University of Turku and Turku University Hospital, Turku, Finland; 6 Clinical Pharmacology Unit, Turku University Hospital, Turku, Finland

**Keywords:** Patient recruitment, Clinical trials, Success factors, Failure, Electronic health records, Nordic countries, Interview, Qualitative

## Abstract

**Background:**

Patient recruitment for clinical trials is challenging—only approximately one third of all trials recruit their participants as planned. The pharmaceutical industry’s views on recruitment success have not been comprehensively investigated, although the industry globally conducts almost one third of all clinical drug trials. This study explored patient recruitment success and failure factors and the role of electronic health records (EHR) in the recruitment of trial participants in the Nordic countries.

**Methods:**

A descriptive qualitative interview study was conducted with 21 representatives of the pharmaceutical industry or contract research organizations operating in Finland, Sweden, Denmark, and Norway. The interviews covered 34 clinical pre-market drug trials. Qualitative data were analyzed using inductive content analysis.

**Results:**

Four main categories were derived to represent both success and failure factors, whereas a fifth category represented only failure factors: (1) sponsor-related (protocol and trial preparation and feasibility evaluations), (2) site/investigator-related (access to patients, motivation, commitment and resources), (3) patient-related recruitment factors (medical need, patients’ role in their care and attitudes towards trials), (4) Sponsor—sites—patients collaboration factors, and (5) start-up related factors. EHR was the most important source of recruitment, utilized in 29 out of 34 trials discussed. Revision of the legislation regulating the secondary use of EHR was highlighted as the most effective measure to facilitate the use of EHR in recruitment of trial participants.

**Conclusions:**

The industry representatives recognized quite well their own role in contributing to the success or failure of the recruitment: to facilitate recruitment of trial participants, many obstacles can be avoided with better trial preparation and proper feasibility evaluations. As access to patients represents one of the key success or failure factors of recruitment, and as the EHR is regarded the main source of searching for and finding patients, the development of EHR utilization appears to represent a powerful tool to improve patient recruitment.

**Supplementary Information:**

The online version contains supplementary material available at 10.1186/s13063-022-06144-9.

## Background

Patient recruitment into clinical trials is a well-recognized challenge all over the world. Common problems encountered in recruitment are a lack of eligible patients, the high burden of trial procedures for patients and investigators, strict eligibility criteria, and lack of resources of the trial staff [1, 2]. Failures in recruitment can delay trials, leading to increased costs and delayed access to new treatments [3]. In the worst-case scenario, recruitment failures may lead to unnecessary patient interventions with investigational drugs without achieving usable study results [4].

Recruitment research with qualitative techniques has concentrated on the investigators’, patients’, or non-industry sponsors’ views, but less is known about the pharmaceutical industry’s perspective. As approximately 27% of all clinical drug trials are industry-sponsored (www.clinicaltrials.gov), it is important to understand also the industry’s views on the topic. The sponsor has ultimate responsibility for all aspects of trial conduct, so in terms of patient recruitment, the sponsor’s guidance and oversight are crucial, even in cases where the sponsor does not have direct contact with the targeted patient population.

The technological transformation of patient records from paper to electronic format has increased substantially over the past two decades [5]. An electronic health record, EHR, is “an electronical format, longitudinal health information on individual patients recorded by health care personnel and to be used in the patient care” [6]. A typical individual EHR may include the patient’s medical history, diagnoses, treatment plans, medication records, and laboratory and other test results [7]. Examples of adoption of information technology in the health sector, including the development of EHR systems, are today commonplace in every World Health Organization’s (WHO) member state in the European region [8]. More than half (59%) of WHO’s European member states reported having a national EHR system, and 69% of those have legislation governing its use [8]. On a global level, the national EHR systems are less common; somewhat less than half (57/125) of the WHO member states who participated in the WHO survey had a national EHR system [5]. All Nordic countries have national EHR systems, covering almost 100% of their generated patient data [9, 10].

The WHO has also encouraged countries to develop their national EHR systems for research purposes [8]. The use of EHR data for research purposes is seen to enhance the transparency of research and to generate improved efficiency of the use of the publicly funded EHR systems. There are many initiatives for EHR secondary use. For example, the European Institute for Innovation through Health Data (i~HD) initiative has worked towards the harmonized secondary use of health data in Europe [11]. i~HD plays a strong role in many national and international R&D projects and European-wide collaborative initiatives, including several IMI projects where academic research groups from all over Europe collaborate with global pharmaceutical companies. In addition, regulatory bodies overseeing the development and use of pharmaceuticals have expressed a positive stance on the use of electronic patient data for enhancing clinical trial conduct and have provided written guidance on their expectations regarding clinical source data existing in electronic format [7, 12–15]. One of the priorities in 2021–2025 of the European Union Commission is the creation of a European Health Data Space (https://ec.europa.eu/health/ehealth/dataspace_en), which is planned to promote better exchange and access to different types of health data, including the secondary use of EHR data. The Nordic countries have long traditions and trusted reputations in conducting clinical trials, in maintaining their hospitals’ patient data in EHR systems [9] and in using clinical quality registers for research purposes [16]. The similarities between the Nordic countries in terms of health care infrastructures and the well-organized public health care systems confer multiple advantages for research, such as comprehensive health care data sources covering the entire population. Unique personal identity numbering systems allow researchers to combine personal information stored in different sources. The Nordic countries are in the front line of the secondary use of EHR data [9, 10] and are increasingly executing new regulations for the secondary use of health data. Examples on these are the Act on the Secondary use of health and social data (552/2019) in Finland and the upcoming revision of the Act on Patient data (currently 2008/355) in Sweden. It was seen as important to assess the current and future roles of EHR data in the recruitment of trial participants, as there is still not sufficient understanding of how the data held by health care systems, especially in the form of EHR, are used or could be used for recruitment into clinical trials. The EHR practices of the Nordic countries are of interest also in other highly developed countries who are considering opening of their EHR systems for purposes of secondary use.

We conducted a qualitative interview of representatives of the pharmaceutical industry and contract research organizations (CROs) operating in the Nordic countries (Finland, Sweden, Denmark, Norway). As two thirds of all clinical drug trials conducted in the Nordic countries are industry-sponsored, targeting the interviews to those countries to obtain the industry’s views on EHR and recruitment was justified. Our study was part of a larger project that investigated the use of EHR for feasibility evaluations, patient identification, and recruitment into clinical trials in the Nordic countries. In this study, we wanted to become acquainted with the pharmaceutical industry’s views on the success or failure of recruitment into clinical drug trials. A special focus was on the use of EHR in the patient recruitment.

## Methods

### Study design

A qualitative descriptive study design was used [17]. The participants were interviewed about their varied, shared, and context-bound experiences [18]. Data were collected using semi-structured interviews [19], which were conducted between 15 March 2019 and 11 July 2019. The interviews lasted 32–90 min and they were held in English (14 interviews) and in Finnish (7 interviews).

### Participants

The interview participants were recruited by email invitations through suggestions of the Nordic Pharma Industry associations, Pharma Industry Finland (PIF, Finland), Läkemedelsindustriföreningen (LIF, Sweden), and Legemiddelindustrien (LMI, Norway). Participants in Denmark were approached through personal industry contacts by MB and NL. NL contacted all potential participants and confirmed their eligibility. Participants were eligible if they were working for a pharmaceutical company or a CRO representing the industry and were involved as sponsors in conducting phase I–III clinical drug trials with patients. Participants were purposively sampled to ensure representation of all four countries and various types of pharmaceutical companies and CROs with regard to size and therapeutic area of expertise. The participants should have exerted an impact on the site identification and/or patient recruitment process in their company, which was confirmed before the interviews. Participants only involved in phase I trials with healthy volunteers were excluded. NL conducted all interviews. NL previously knew two of the participants.

The inclusion of participants was continued consecutively until saturation was reached, i.e., until no new meanings to the categories were captured from the interviews [20]. After the first 11 interviews, an overall description of the phenomena under investigation started to form, and no surprising or new items were identified during the subsequent interviews #12–21. After 21 interviews, we were able to clearly define the different elements of recruitment success and failure and the role of EHR in the process. Based on this, we concluded that saturation had been reached after 21 interviews. The process could also be confirmed afterwards by reviewing the diary notes taken during the interviews.

Twenty-eight interviewee candidates were contacted; one refused to participate, and three candidates did not respond to the email request and another three did not fulfill the inclusion criteria (conducted trials only with healthy volunteers). Of the 21 participants, all were senior-level employees having an impact on the site identification and patient recruitment process; seven were from Finland, five from Sweden, five from Denmark, and four from Norway. The participants’ characteristics are presented in Table 1.

**Table 1:** Participants’ professional background and clinical trials experience

Participant characteristics	***N*** = 21
Country	
Finland	7
Sweden	5
Denmark	5
Norway	4
Position in the company	
Clinical Study Management	6
Clinical Operations	6
Clinical Site Management	4
Feasibility and Recruitment Management	5
Gender	
Female	17
Male	4
Company type	
Pharmaceutical company	17
CRO	4
Served current employer	
0–5 years	6
6–10 years	7
11–20 years	6
> 20 years	2
History with clinical trials	
< 10 years	2
10–20 years	9
> 20 years	10

### Interview guide and data collection

An interview guide was developed jointly by the researchers NL, MS, and RH, and the open-ended questions were modified multiple times before the final version. If needed to enhance the understanding of the phenomenon, additional questions emerging from the dialog between the interviewer and the interviewee were also allowed. The interview guide (Additional file 1) contained four categorical questions and eight open questions as well as a place for the participants’ notes for preparing for the interview. The guide was tested with one pilot interview, which was included in the analysis, as no major modifications were made after the piloting.

Participants received the questions approximately 1 week in advance. Prior to the interview, the participants were asked to select two phase I–III clinical drug trials that were the most important for their companies, in terms of strategic importance for their business, where they had been involved in 2015–2018 (i.e., started < 4 years before the interviews in 2019), and where at least one Nordic country was involved. No requirements were set to select the trials based on the outcome (success or failure) of recruitment.

*"I chose this one, because it was important to get this first phase I trial to our country. We tried to get it here for so long." (ID 15)*

Ultimately, 34 trials were discussed in the interviews, because seven trials selected by the participants did not meet the above criteria (for example, the trial had not yet started) and one participant had only chosen one trial.

In the interviews, the order of the questions was followed, and the participant’s first trial was discussed thoroughly before the same set of questions was asked about the second trial of that participant. The planned numbers and the actual numbers of the recruited patients were collected for each Nordic country. Thereafter, all participants were asked to judge whether recruitment into their trials in the Nordic countries had succeeded or failed (for ongoing recruitments; on schedule or delayed). If the number of patients recruited in the Nordic countries reached the planned number (a 95% value of the target was allowed), the recruitment was regarded successful. It was possible to compensate for reduced numbers in one Nordic country by exceeding the initial target in another country. If the recruitment target had not been reached or had been reached only after a prolongation of the recruitment period, the recruitment was classified as failed.

The respondents were asked to identify key factors for the recruitment success or failure; the recruitment success factors were requested to be identified from trials perceived as successful by the respondents. Similarly, the respondents were asked to describe recruitment failure factors from trials regarded as failed in recruitment.

For the trials that failed in recruitment, the participants were also asked to evaluate the recruitment’s contribution to a trial delay, compared to other possible delay factors (on a scale from 1 = “patient recruitment had no effect on trial delays” to 4 = “patient recruitment was the major trial-delaying factor”).

The information on recruitment success/failure, trial schedule, and information on where the sites found the trial subjects were collected from all trials. This numerical information could be used as supportive information to the qualitative analysis.

### Data handling and analysis

The trial profiles and the responses to the categorical questions were collected using REDCap, version 9.1.12 data management software [21, 22], and analyzed descriptively. All other interview contents were transcribed verbatim and managed with Nvivo software, version 12 plus (QSR International Inc., USA). The COREQ checklist for qualitative studies was used in reporting [23].

Inductive content analysis [24] was applied, i.e., the data analysis was not performed according to any pre-determined categories derived from the literature, as there was no proper theoretical framework for presenting the trial sponsors’ views on the recruitment and/or the EHR use. All transcripts were read multiple times to achieve an overall impression of their content. Only the manifest content of the interviews was analyzed. The data were coded by NL. The interpretation of the interviews’ contents, the coding, and the categorizations were thoroughly discussed with and deliberated by another researcher (AA), already at the start of the analysis. The coding process was initiated by identifying factors impacting (positively or negatively) on the recruitment. Thereafter, the sub-categories started to form as similarities and differences in the codes were noted. The codes were collated to sub-categories that were further grouped and abstracted into categories by classifying them into items having similarities or conjunctive causes. The categorization, as derived from the data, was made together with AA, in order to develop a mutual understanding of the meanings of the codes. The role and potential use of EHR in patient recruitment were coded similarly and are reported here under section “Site/investigator-related recruitment factors”.

## Results

### Trial demographics

Each Nordic country appeared in at least half of the trials covered by the interviews: out of 34 trials, Finland, as a country, was involved in 21 trials (62 %), Sweden in 20 trials (59 %), Denmark in 19 trials (56 %), and Norway in 17 trials (50 %). Most of the discussed trials involved adult patients (91 %) and were phase III trials (65 %). In all trials, 90 % of the planned patients had been recruited (6249/6931 patients). In successfully recruited trials, 98 % of the patients had been recruited (5445/5573), whereas in unsuccessfully recruited trials 59 % of the planned patients had been recruited (804/1358) (Table 2). Of the 34 trials, 13 trials were delayed from the original schedule. The interview participants evaluated that patient recruitment was the major reason for the delays in nine trials, whereas in four trials the recruitment had no impact on the trial delays. In those cases, the reasons were slow ethics committee procedures (one trial), slow start by the sites (two trials), and delayed availability of the investigational medicinal product (one trial).

*Q: “If you compare the delays caused by recruitment to other study delays…”*

*A: “The main reason was the patient recruitment. Of course, there may have been other delays like related to the supplies etc., but it was nothing compared to the patient recruitment. Difficult recruitment was driving everything in this trial.” (ID 11)*

Other details of the trials’ characteristics are listed in Table 2.

**Table 2:** Trial demographics

Trial demographics, ***n*** = 34 trials		
Phase	Phase I or Phase II	Phase III
	12 (35 %)	22 (65 %)
**Patient type**		
Adults	10	21
Pediatric patients	2	1
**Therapeutic area**		
Oncology	4	4
Neurology	3	3
Endocrinology	1	4
Other ^a^	4	11
**Recruitment status**		
Recruitment ongoing	4	8
Recruitment ended	8	14
**Trial status**		
Trial ongoing	5	10
Trial completed	7	12
**Trial schedule**		
Trial on schedule	7	14
Trial delayed	5	8
**Recruitment outcome**		
Success	10	7
Failure	2	15

^a^ Other: Cardiology, gastroenterology, pulmonology, psychiatry, nephrology, dermatology, and immunology

Half of the trials (17/34) discussed in the interviews had succeeded in recruitment, whereas the other half had failed to meet their initial recruitment timelines, as judged by the respondents. In five interviews, both of the selected trials had succeeded whereas in four interviews both trials had failed in their recruitment. In six interviews, one trial had failed and the other had succeeded in recruitment, and in four interviews, only one trial was discussed, either succeeded or failed in its recruitment.

Recruitment failures most commonly occurred in phase III trials; only one third of the discussed phase III trials succeeded in recruitment, while 10 out of the 12 discussed phase I and II trials succeeded to recruit as planned (Table 2).

### Success and failure factors of recruitment

Four main categories (sponsor-related, site/investigator-related, patient-related, sponsor-site-patient collaboration related factors) were evident from the data representing both success and failure factors, whereas the fifth category (factors related to start-up activities) was noted only among failure factors. Almost all success factors of recruitment were mostly opposite to the failure factors. Table 3 lists the main categories and categories for key recruitment factors as perceived by the respondents.

**Table 3:** Success and failure factors of patient recruitment in clinical drug trials

Sponsor related	Site/investigator related	Patient related	Collaboration related	Start-up related^**a**^
Trial protocol	Access to patients	Patients’ medical need for new treatments	Sponsor-site-patient collaboration	Ethics committee evaluation
Trial preparation and feasibility evaluations	Investigators’ motivation for trials and commitment to recruitment	Patients’ role in their care and attitudes to clinical trials		Site contracts
	Site resources, setup, and experience			

^a^ Start-up was only identified as a recruitment failure factor

#### Sponsor-related recruitment factors

##### Trial protocol

A clear and easy-to-conduct protocol for both investigators and patients was seen to contribute to successful recruitment. For example, combining trial visits with regular patient care visits was considered to minimize the burden for the patients, whereas many invasive study procedures or a need to be absent from work often emerged as patient-related obstacles for recruitment. In addition, the respondents highlighted that carefully considered and clear inclusion and exclusion criteria facilitated recruitment, whereas too strict or ambiguous criteria contributed to recruitment failures.

*"…And also the lightness of the protocol: Taking into account this was a phase II trial, it was relatively light to conduct the study. The sites gave feedback that we really had thought about the patient. The study visits were synchronized as much as possible with the normal control visits not to burden the patient too much. The investigators liked it very much and they were willing to take on also more patients." (ID 7)*

##### Trial preparation and feasibility evaluations

Some participants highlighted that careful preparation of the trials and a thorough conduct of the feasibility evaluations remarkably improved the success of recruitment. If there had been proper evaluations before trial start, sponsors were able to select the most appropriate countries and sites for their trials. They were able to define realistic recruitment targets and to minimize the impact of several factors that could potentially adversely affect recruitment, such as competing trials by other sponsors and the paucity of potential trial subjects. Complementing these findings, the participants reported multiple cases where inadequate trial preparation and feasibility evaluations had contributed to failed recruitment. For example, too unrealistic estimations about the number of patients had been set, or the patient population available was simply too small because of competitive trials, which had not been considered during the feasibility evaluation.

*"When the recruitment started, it was actually a surprise that the population was that small in European countries." (ID 12)*

*"It was very difficult to recruit, mainly because it was a highly competitive area. And because of the small population. There were many trials from different companies ongoing almost at the same time." (ID 11)*

In some cases, unsuitable sites had been selected; the typical examples were sites not treating patients who would be eligible for the trial, relating to the severity of the disease. The patients were in some cases too ill to meet the selection criteria, or they were so well treated that they did not qualify for the trial, resulting in a lack of treatment-naïve patients for example in diabetes or asthma trials.

#### Site/investigator-related recruitment factors

##### Access to patients

The participants perceived that sites having a clear understanding of their own patient population and having access to it contributed to successful recruitment.

*"The sites could use their electronic records to track whether they have such patients who fulfill these criteria. That was quite easy for the sites who already had that population." (ID 9)*

Correspondingly, one of the main reasons for poor recruitment was the lack of patients. The root cause for this was mainly that there had been a poor feasibility evaluation, as mentioned above in section “Sponsor-related recruitment factors”. The participants also reported a few cases where the National Coordinating Investigator (NCI) was reluctant about the use of new digital recruitment methods, such as social media or web-based recruitment tools, even when recruitment from the site’s own patients was not sufficient. This also affected the other investigators’ attitudes on using new recruitment methods for improving the access to patients.

##### Role of EHR in access to patients

When the participants were asked how trial sites identified their trial patients, site EHRs emerged as the most important source, used in 29 out of 34 trials (Table 4).

**Table 4:** Reported sources of potential trial subjects, *n* = 34 trials

From where did the investigators find trial participants?	Number of trials
Site’s own patients in their EHR	29 (85 %)
Referrals within/from outside of the hospital	9 (26 %)
Patient register or biobank	6 (18 %)
Social media (Facebook, etc.) and web-based recruitment tools	5 (15 %)
Advertisements in newspapers and magazines	5 (15 %)
Patient organizations (advertisements, public lectures)	5 (15 %)

*"In the EHR, of course, there they found most of the patients, but Facebook was the second most effective." (ID 23)*

*"But I still think that falling back on the primary patient flow in the clinic in collaboration with other physicians is the most successful way to enroll patients in most studies. A very hands-on approach at the local level, using the local study team in the hospital as a unit to drive the study, still by far remains the most effective way to run the study." (ID 5)*

Extensive variation was reported in how EHRs were used: Some sites “knew” the patients they have in their EHR and recruited them without any further searching from the EHR, whereas some sites searched their entire hospital’s EHR data by filtering with certain eligibility criteria with the help of the hospital’s IT department.

The role of other tools, such as referrals within/from outside of the hospital, patient registers, patient organizations, social media, web-based recruitment tools, and/or traditional advertisements, were much less frequently used than EHR. They were most commonly used along with the EHR, and often only after realizing that the recruitment target would not be reached solely with the site’s own patients. For example, only six trials used patient registers, such as national cancer registers or registers for diabetes, cardiac diseases, or biopsies and for a chronic, rare, progressive disease. However, most of them were regarded as useful for finding potential trial subjects in those few trials.

*"It was not a big effort to find these patients also because we have registers for this disease in the Nordic countries." (ID 1)*

*"Sweden was the only country who embraced new technologies. They on their own initiative created a web-based recruitment campaign. And we really tried to speak it up in other countries, but only Sweden had the courage to explore this opportunity." (ID 2)*

A main benefit of using the EHR was the fast identification of potential trial subjects. Some participants also highlighted that the extensive use of EHRs in searching increases the patients’ opportunities to equally join in clinical trials and to gain access to new treatment opportunities. However, some participants also mentioned that there are disease entities that are not adequately identified in the EHR, and in such cases, other recruitment methods, like the ones described above, are better suited for the purpose.

According to some of the participants, Nordic sites use EHRs and patient registers in their recruitment more often than sites located in other European countries.

*"Also here we can see that we (the Nordic countries) can more easily use these electronic capabilities (for recruitment). Finland and Norway pave the way. If we think of other countries (outside of the Nordics), they still use these rather traditional methods. And because of that, we are often quicker (recruiting in the Nordics than in other countries)." (ID 3)*

The participants stated that due to legislative barriers, the use of EHRs in patient identification and recruitment was not equal in all Nordic countries. Some sites were able to use their EHR system more flexibly; for example, Finnish sites could take advantage of hospital-based biobanks when contacting potential trial subjects found with the EHR search, to inquire about their willingness to participate. Interpretations of data protection legislation were also seen to hinder hospitals from sharing their EHR data with other sites located in the same region for the purpose of searching for potential trial subjects. A balance between the legislation and the research needs was highlighted in many interviews.

*"My biggest wish would be to see standardized electronic health system per country, where they actually can pull data in a good GDPR way to identify patients." (ID 9)*

Most participants emphasized that the need for patients’ EHR data will become ever more important in the future, especially in the recruitment into trials to treat rare diseases and into trials on targeted medicines.

*"It is absolutely necessary for the investigators to search from hospital EHR to search patients but perhaps it can be used in more structured way in the future." (ID 20)*

The major benefit for the whole Nordic area was seen in larger entities than single hospitals, involving even Nordic-wide EHR data lakes.

*"EHR can definitely do a lot (in recruitment). I don’t know why there is such a problem that the Nordics are not seen as one region. I know we have talked about it. I guess one of the reasons is that the laws (regulating the EHR use) are so different." (ID 13)*

The participants shared a concern on the quality of the EHR data; the information contained in the systems should always be up-to-date, in a structured, searchable format, and comprehensive in data variables and cover a sufficient number of patients in order to be really helpful in finding potential trial subjects.

##### Investigators’ motivation for trials and commitment to recruitment

When the investigators were motivated, had a passion for the research topic in question, and were committed to recruitment, they were able to enroll patients as planned—even if it was not always easy.

*Q: “What do you think these Swedish sites did differently?”*

*A: “I think they have some very, very dedicated investigators who were deeply involved in this disease.” (ID 11)*

Investigators’ experience of the investigational drug from earlier trials or a belief in the drug’s potential benefit for patients influenced the recruitment success. Some investigators lost their motivation during the recruitment period due to repeated screening failures, i.e., patients were identified, provided consent, and screened for the trial but were subsequently found not to be eligible.

*"Some (investigators) thought it was too much work and too much time spent. Even though they were reimbursed for all the time they were using." (ID 22)*

In some cases, the lack of investigator’s motivation became apparent when the investigator was not devoting enough time for the conduct of the trial or when he or she was not actively offering trial participation for his or her patients. According to one respondent, even an additional fee that the sponsor paid for patient pre-screening activities was not helpful when the investigators were not motivated.

*"During the visits, we requested them (the investigators) to perform a pre-screening of patients in the hospital’s EHR and offered pay an additional fee for that work. But the work was never done." (ID 18)*

The participants noted that most sites recruiting successfully were committed to recruitment; for example, by having a sub-investigator purely dedicated to recruitment tasks or by pre-screening potential subjects by a study nurse. Furthermore, sufficient preparations for recruitment, well in advance, enhanced the recruitment efficiency.

According to the interview participants, some investigators did not have enough “hunger” or ambition to conduct trials, even if they had agreed to join. Some investigators’ attitudes were considered as blasé and not taking seriously the recruitment targets set for their sites.

*"If we take the highest enrolling countries (of our trial), Poland, South Africa, Israel, Turkey, I think the motivation at the sites was much bigger than in Scandinavia. It was my impression that Scandinavian sites were somewhat over-confident (by thinking) ok, if we don’t succeed in this study, we will succeed in the next one. Whereas in these other countries, they don’t feel confident that they will get the next study, so they will have to deliver in this study." (ID 20)*

##### Site resources, setup, and experience

Well-organized site processes and sufficient staff resources for trial conduct enhanced successful recruitment. Particularly, dedicated clinical trials units within hospitals were mentioned by the participants. Competent and experienced site personnel and the delegation of trial responsibilities to sub-investigators and study nurses were also seen as reasons for successful recruitment. The participants correspondingly reported that if these were missing, then they were reasons for poor recruitment.

Participants experienced that some Nordic hospitals did not recognize the competitive environment where trial sites and countries should conduct industry-sponsored clinical trials: Investigators were not always provided with sufficient time to conduct the agreed research appropriately, and hospital bureaucratic procedures were in some cases slow and complicated, thus undermining subject recruitment efforts.

*"And the best performer has been, I guess, Denmark, it is a research-friendly country.. it is really good, they have very good systems.. and they have the resources, which I think are lacking at some other sites, who do not have study nurses who could pre-screen their patients…They (Danish sites) can do research during their daily working time, whereas in our country, it cannot be done in many hospitals." (ID 18)*

#### Patient-related recruitment factors

##### Patients’ medical need for new treatments

Most participants cited that when there was a clear unmet need for the new drug treatment, trial subjects were found easily and recruitment was successful. However, in some therapeutic areas, patients did not feel any need to participate in a clinical trial; they felt that they were already receiving the best possible treatment for their disease.

*"In this disease, there is an unmet need for medical treatments. The patients are very much interested in participating in trials." (ID 24)*

##### Patients’ role in their care and attitudes to clinical trials

Nordic patients were perceived as active in their own care and treatment, and willing to join clinical trials as study subjects. The respondents also noted that patients trust the Nordic sites as care providers, which was seen as one recruitment success factor.

*"…And they (Nordic sites) do have the good reputation also. Patients know that if they are be treated there, they will be well taken care of." (ID 16)*

However, in some trials, the sites did not find suitable trial participants because their patients had not yet formally received the required diagnosis, but only had an increased risk of having the disease in question, or the sites failed in recruiting patients because of the patients’ lack of awareness of their disease, attributable to a denial of its presence.

*"These patients were not necessarily identified by themselves as having asthma. Mild asthma is something people can ignore until it becomes more severe." (ID 13)*

#### Collaboration between sponsors, sites, and patients from the recruitment perspective

##### Sponsor-site collaboration

Collaboration, communication, and building of mutual trust among sponsors, sites, and patients were seen as a crucial success factor in recruitment, whereas a lack of collaboration between the different stakeholders slowed down the recruitment. For example as stated by the participants, many changes in the Clinical Research Associates (CRAs) led to misunderstandings and breakdowns in the communication between the sponsor and the sites, whereas an experienced, permanent CRA and regular monitoring visits contributed to recruitment success.

*Q: “What do you think in this trial was the most important thing for the success of the recruitment?”*

*A: “We had even more close contact with the sites.” (ID 21)*

##### Site-site collaboration

Recruitment performance was also seen as often being poor in cases when the sites did not collaborate with each other by sharing experiences or valuable trial-related information. Recruitment was seen to benefit from having investigators who had good networks of colleagues within and outside of their own hospital. A National Coordinating Investigator (NCI) with effective information exchange and collaboration was considered to improve the other investigators’ recruitment success.

*"But they (site personnel) are working very well with the recruitment and they are contacting the other clinics in the region, so they have extensive communication with other clinics. Communication is working really well for that site. So they are looking outside their own hospital to find patients and that´s what I think is the key here." (ID 1)*

##### Site-patient collaboration

One example of how site-patient collaboration was seen to contribute to the recruitment success was the personnel’s practice of providing adequate information to their patients about the trial procedures. Some respondents highlighted the importance of contacts with patient representatives via patient organizations already during protocol development. This type of patient collaboration was seen as very valuable for successful recruitment.

*“I think the reason for success is related to the relation-building activities.. It is very much about speed of communication and relations.” (ID 11)*

#### Start-up related failure factors in recruitment

##### Ethics committee evaluations and site contracts

The participants estimated that slow and unclear ethics committee handling procedures were one of the reasons for recruitment failure. Many participants mentioned that after adoption of the European Union’s (EU) General Data Protection Regulation 679/2016 (GDPR), the situation worsened because of the different and unpredictable GDPR interpretations made by the ethics committees. GDPR is a data privacy and security law placing obligations on all organizations who target or collect data related to people in the EU. Delayed trial start shortened the recruitment period for some Nordic countries, which in some trials was a reason for not reaching the planned recruitment target.

*"But then GDPR changed things and the Danish Ethics committee rejected the study. And for that reason, they (the site) had to re-apply and study start was delayed. Then, recruitment ended prematurely, because it was globally competitive, and for that reason we did not reach those numbers…So the interpretation of the GDPR affected the trial in Denmark, and also in Finland it caused a delay. But in Sweden it did not, I guess because there they had quite clear instructions from the authorities about how data protection should be evaluated (by the Ethics committees)." (ID 18)*

Additionally, a few respondents mentioned that slow site start-up performance, most commonly perceived to result from a time-consuming contract process, shortened the recruitment period for such sites.

## Discussion

### Recruitment success and failure

Of the trials that the participants had selected for the interviews, one half succeeded in recruitment and the other half failed (17 out of 34). The success of recruitment was more frequent in phase I and II trials, whereas recruitment failures were common in phase III trials. As the small numbers of trials covered and the qualitative design of this study do not allow us to make a wider generalization, quantitative investigations will be needed to confirm whether recruitment of patients into phase I and II trials in the Nordic countries is more successful than elsewhere.

Recruitment success and failure factors in the Nordics have not been investigated earlier. The results show similarities with earlier results obtained from other industrialized countries, thus strengthening the results reported by earlier studies. Poor patient recruitment has been reported as the main reason for delayed trials [1, 3]**.** Most recruitment failures in the trials covered by this study occurred in phase III trials. As Nordic patients constitute a relatively small proportion of the total number of patients in multinational phase III trials, the recruitment difficulties of Nordic sites as such will not necessarily contribute to trial delays on a global level. However, poor recruitment performance will affect the site’s possibilities to participate in future new trials. Failed recruitment will be evident in the performance databases when sponsors are selecting sites for new trials. In countries with small populations, this may have considerable consequences for the entire country, reducing their opportunities to take part in new clinical trials.

As we focused on the perspective from the pharmaceutical industry and CROs, success and failure factors linked with sponsor-related items now received more attention than usually in previous research on recruitment factors. Especially the impact of poor feasibility evaluations and trial planning to recruitment success is more highlighted here than in the literature reporting investigators’ or study nurses’ views [25]. Some protocol-related factors have been reported earlier, for example by Briel et al., who noted that trials with poor recruitment often had more stringent eligibility criteria, caused a higher burden for patients and investigators, or more often contained outdated control interventions than trials with successful recruitment [2]. Interestingly, they also reported that the type of sponsorship influenced recruitment success: randomized clinical trials with poor recruitment were often investigator-sponsored whereas similar randomized clinical trials without poor recruitment were often industry-sponsored. The poorer recruitment in the investigator-sponsored trials was explained by less professional trial organizations and limited funding. In our study, none of the industry sponsors mentioned these factors as contributors to the success or failure of recruitment. In addition to non-industry sponsorship, a greater number of eligibility criteria, poorer control interventions, and fewer trial sites have been reported to be associated with failed recruitment [26]. In our study, we also captured the sponsors’ positive experiences on well-designed and well-written protocols and their recognized influence on recruitment. Our study results strengthen the view that the protocol-related factors potentially affecting recruitment should be evaluated already during trial planning. All burdensome factors contributing to recruitment failure cannot or should not be avoided (such as masking of the treatments or some invasive trial procedures). Nevertheless, sponsors should carefully evaluate what is necessary for the trial, and whether there are alternative ways to conduct the trial. The conduct of feasibility evaluations should also be improved.

The investigator/site-related recruitment factors identified in this study were similar to those already reported by others [25, 26]. Examples of these were the investigators’ motivation, resources, and experience. However, the investigators’ intellectual and emotional challenges in recruitment that arise when combining research with clinical tasks were not mentioned by our respondents. The lack of suitable patients has been highlighted earlier as the most important reason for failed recruitment [25, 27], and this was also evident in our study. Also factors related to collaboration between all stakeholders were in line with earlier findings [28].

Patients’ strong trust in their care providers and favorable attitudes towards clinical trials were recognized as success factors. Nordic patients are known for their commitment to trials after joining them [29]; retention of enrolled patients in trials with a long duration may be a lesser concern in the Nordics than elsewhere [28, 30]. Still, this study only reflects the sponsors’ views on patient preferences and attitudes, and further empirical research among Nordic patient populations would be needed to gain a better understanding of all patient-related recruitment success and failure factors.

Another interesting feature in our study was the impact of slow start-up procedures on recruitment success, not commonly reported in previous literature. As the pharmaceutical industry is conducting trials in multiple countries and continents, its representatives may have a better view of the differences in start-up times than stakeholders working on a local level. However, this finding supports the conclusions of Cheng et al. who investigated non-commercial cancer trials sponsored by the National Cancer Institute Cancer Therapy Evaluation Program, in the USA. They stated that trials with longer development times (from letter of intent to the start of recruitment) were significantly less likely to achieve their accrual goals than trials with a more rapid start-up [31].

Lastly, a clear connection was noted between the observed recruitment factors. For example, the complex protocol and poor feasibility performed by the sponsor reflected on the recruitment factors at the level of the sites/investigators; selection of inappropriate sites lacking suitable patients or sufficient resources were often connected to investigator’s motivation and commitment. In turn, this influenced how often the investigators offered the trials to the potential trial subjects and how much they collaborated with patients, with other sites and with the sponsors. The industry representatives were well aware of this chain and their own role in contributing to the success or failure of the recruitment process. We did not find this factor reported in trials with other types of sponsorship.

### EHR in patient recruitment

EHRs were the most important source for recruitment, used in 85 % of the trials covered by the interviews. Patient registers, social media, and other sources had been used surprisingly rarely in the trials discussed. For example, the patient registers were only used in 6 out of 34 trials, despite the existence of comprehensive nationwide patient registers in some of the Nordic countries. It appears that the use of multiple recruitment methods could be combined to increase recruitment success [30].

Some respondents stated that Nordic sites use EHRs and other patient registers in their recruitment more commonly than sites located in other European countries. There is known to be extensive variation in the secondary use of EHRs globally, as well as in data quality and the time required for obtaining EHR data in different countries [32], but determining the exact usage patterns of EHRs in trial subject recruitment between different countries remains for future work. However, according to a report investigating 87 globally conducted clinical trials, only 11 % of the trials used EHRs in recruitment [33], which is in stark contrast with our results.

Even if EHRs were widely used for recruitment in the trials of this study, the respondents highlighted also the limitations. Legislative constraints and differences between the Nordic countries in data protection legislation and its interpretation were considered as prohibiting the more extensive use of EHRs in patient recruitment; this could include their use within hospital departments or between the hospitals in the same region or nationally. In order to facilitate the secondary use of EHR data for clinical trials, the legislation should be developed by taking into account all stakeholders’ perspectives. It is important to safeguard the patients’ rights for protection of privacy, while opening the use of EHR data for research. For example, allowing access to the patient data in aggregated format should be considered. A robust infrastructure of policies, standards, and best practices for the secondary use of EHR data [34] and requirements for clarifying the legislation regulating its secondary use have been presented [35]. For example, Bahr and Schlünder suggested a code of practice for EHR secondary use in Europe. The purpose of this code of practice would be to resolve issues in a way that would balance the need to make research possible and the need to protect the patients’ privacy at the same time.

The need for wider utilization of individual health data in health-related research has been well recognized [10, 36], and it also emerged in this study. Overall, the respondents’ views on the EHR secondary use in clinical trial recruitment were very positive. They found it becoming more important in the future because of personalized medicine and because other, more patient-centric approaches will also change future clinical trials. Based on the respondents’ views, it appears that small countries, such as the Nordic countries, should try to combine their EHR data into larger nationwide or even pan-Nordic entities. Ideally, the Nordic countries would then be considered as one region, especially for trials in rare diseases. At best, it might become possible to plan trials where patients would travel from one Nordic country to another for at least some of the trial visits [29]. Some Nordic research networks, e.g., NordicNect (https://nordicnect.org/), already facilitate such cooperation in early-phase cancer trials [37].

Based on our results, EHR data are very important source for the recruitment of trial participants. There is also evidence of a slight improvement in the recruitment success of trials: the number of trials reaching or exceeding their enrolment goals has slightly increased from 2012 to 2020 [33]. It is possible that this apparent improvement is due to the implementation of novel recruitment methods, such as EHR queries and other digital solutions.

As access to patients is one of the key recruitment success factors, and with EHR being the most frequently used source searching clinical trial participants, it follows that the development of EHR utilization appears to represent an important tool to improve patient recruitment. In addition, the quality (contemporaneousness, searchability, comprehensiveness in data variables and number of patients) of the EHR data is of utmost importance for being able to identify suitable trial participant candidates in the EHR in an efficient and effective manner [38]. At the same time, stakeholders should work towards addressing public concerns regarding EHR secondary use. The methods for searching EHRs, for assessing eligibility and assessments of the accuracy of patient identification, should be documented and reported adequately [39]. This would, hopefully, increase the transparency and the public acceptance of the EHR secondary use.

## Study strengths and limitations

As there is more published research on recruitment failure factors than on success factors [30], we wanted to investigate the factors influencing recruitment from both directions: recruitment success factors in successfully recruiting trials and failure factors in trials that failed in their recruitment. Nonetheless, when viewing the situation from both directions, we identified the same four main categories, which strengthens our belief that the key recruitment factors were indeed identified in this qualitative investigation. The amount of data obtained in the interviews was extensive and provided an in-depth analysis of the current situation as viewed by industry representatives. Using the research questions (recruitment success/failure) as an initial coding frame may have slightly limited the inductive approach of our analysis.

We conducted this study in four Nordic countries, as they have very similar cultural features and health care systems. In addition, their economies and living standards are rather similar, with equivalent influences on disease prevalence and outcomes, quality of care and inhabitants’ possibilities to receive health care and treatments. These countries, with their total of 27 million inhabitants, constitute an important region for conducting clinical trials. Therefore, we believe that it was advantageous that this study was conducted across the national borders of this geographical region, representing highly developed societies with well-functioning public health care systems and national EHR systems.

The trial selection criteria were defined a priori and it was up to the respondents to select which of their trials they would discuss in the interviews. This may be viewed as a possible limiting factor in the study. To minimize that, all respondents were instructed to choose their trials in the same way by one researcher (NL). For example, the term “important” did not refer to a trial they as individuals liked best but to a trial which was strategically important for their company from a business perspective.

Investigating the impact of the investigators’ compensation to recruitment success would have been interesting but was outside of the focus of the current study. Future studies could usefully explore the impact of compensation on recruitment success. One researcher (NL), with a long background in the pharmaceutical industry, performed the coding of the data. The possible impact of the researcher’s own perceptions was minimized by following a pre-defined interview guide with all participants. Coding decisions, categorization, and all interpretations were evaluated and debated in systematic discussions with another experienced researcher (AA) from the very beginning of the analysis process.

## Conclusions

Success in patient recruitment can be traced to long before the first patient is recruited into the trial, already during the trial’s planning phase. Factors influencing to the recruitment seem to pertain to each other. Recruitment success is dependent on multiple factors, and all trial stakeholders have important impacts; sponsors, investigators/sites, and patients. In addition, trial start-up procedures (Ethics committee evaluation and site contracting) should be streamlined and facilitated in order to promote success in clinical trial recruitment. The same factors that favor successful recruitment may jeopardize it and lead to its failure.

Access to patients is a key success factor for recruitment, and the EHR is an important tool when searching for potential trial subjects. Therefore, the development of EHR utilization appears to represent a powerful tool to impact on the patient recruitment. The legislation on the secondary use of EHR data for patient recruitment should aim for transparent processes in order to create mutual trust among all stakeholders.

## Supplementary Information

**Additional file 1.**

## Data Availability

The Interview guide is displayed in Additional file 1. The datasets analyzed are not publicly available to ensure the privacy of the interview participants and their companies but are available in a blinded fashion from the corresponding author on reasonable request.

## Abbreviations

CNS: Central nervous system
CRA: Clinical Research Associate
CRO: Contract Research Organization
EHR: Electronic Health Record
EU: European Union
GDPR: General Data Protection Regulation (EU)
ICF: Informed Consent Form
IT: Information Technology
LIF: Läkemedelsindustriföreningen (Sweden)
LMI: Legemiddelindustrien (Norway)
NCI: National Coordinating Investigator
PIF: Pharma Industry Finland (Finland)
WHO: World Health Organization

## Glossary

Sponsor^1^: An individual, company, institution, or organization which takes responsibility for the initiation, management, and/or financing of a clinical trial. (ICH GCP 1.53.)
Investigator^1^: A person responsible for the conduct of the clinical trial at a trial site. If a trial is conducted by a team of individuals at a trial site, the investigator is the responsible leader of the team and may be called the principal investigator (ICH GCP 1.34.)
Protocol^1^: A document that describes the objective(s), design, methodology, statistical considerations, and organization of a trial. The protocol usually also gives the background and rationale for the trial, but these could be provided in other protocol referenced documents. (ICH GCP 1.44.)
CRA^1^: Clinical Research Associate (the Monitor) is representing the trial sponsor and is responsible for overseeing the progress of a clinical trial, and of ensuring that it is conducted, recorded, and reported in accordance with the protocol, Standard Operating Procedures, Good Clinical Practice, and the applicable regulatory requirement(s).
Phase I trials^2^: Study Participants: 20 to 100 healthy volunteers or people with the disease/condition.: Duration of Study: Several months: Purpose: Safety and dosage
Phase II trial^2^: Study Participants: Up to several hundred people with the disease/condition.: Duration of Study: Several months to 2 years: Purpose: Efficacy and side effects
Phase III trial^2^: Study Participants: 300 to 3000 volunteers who have the disease or condition: Duration of Study: Typically 1 to 4 years: Purpose: Efficacy and monitoring of adverse reactions

## References

[1] Hanson LC, Bull J, Wessell K, Massie L, Bennett RE, Kutner JS, Aziz NM, Abernethy A (2014). Strategies to support recruitment of patients with life-limiting illness for research: the Palliative Care Research Cooperative Group. J Pain Symp Manage.

[2] Briel M, Speich B, Von Elm E, Gloy V (2019). Comparison of randomized controlled trials discontinued or revised for poor recruitment and completed trials with the same research question: A matched qualitative study. Trials.

[3] Watson JM, Torgerson DJ (2006). Increasing recruitment to randomised trials: a review of randomised controlled trials. BMC Med Res Methodol.

[4] Treweek S, Lockhart P, Pitkethly M, Cook JA, Kjeldstrøm M, Johansen M (2013). Methods to improve recruitment to randomised controlled trials: Cochrane systematic review and meta-analysis. BMJ Open.

[5] World Health Organization. Global diffusion of eHealth: making universal health coverage achievable: report of the third global survey on eHealth. 2016.

[6] Häyrinen K, Saranto K, Nykänen P (2008). Definition, structure, content, use and impacts of electronic health records: a review of the research literature. Int J Med Inform.

[7] FDA. Use of Electronic Health Record Data in Clinical Investigations Guidance for Industry. 2018. https://www.fda.gov/Drugs/GuidanceComplianceRegulatoryInformation/Guidances/default.htmand/. Accessed 29 Apr 2019.

[8] World Health Organization. From Innovation to Implementation. EHealth in the WHO European Region. 2016. http://www.euro.who.int/en/ehealth. Accessed 30 June 2020.

[9] Bonomi S (2016). The electronic health record: a comparison of some European countries. Lecture Notes in Information Systems and Organisation.

[10] Nordforsk. A Vision of a Nordic Secure Digital Infrastructure for Health Data: The Nordic Commons. 2019. www.nordforsk.org. Accessed 26 Nov 2020.

[11] Kalra D, Stroetmann V, Sundgren M, Dupont D, Schlünder I, Thienpont G, Coorevits P, de Moor G (2017). The European Institute for Innovation through Health Data. Learn Health Syst.

[12] EMA. Reflection Paper on Expectations for Electronic Source Data and Data Transcribed to Electronic Data Collection Tools in Clinical Trials. 2010. www.ema.europa.eu. Accessed 18 Jan 2021.

[13] FDA. Guidance for Industry Electronic Source Data in Clinical Investigations. 2013. http://www.fda.gov/Drugs/GuidanceComplianceRegulatoryInformation/Guidances/default.htm. Accessed 18 Jan 2021.

[14] MHRA. MHRA Position Statement and Guidance Electronic Health Records. 2015. https://assets.publishing.service.gov.uk/government/uploads/system/uploads/attachment_data/file/470228/Electronic_Health_Records_MHRA_Position_Statement.pdf. Accessed 18 Jan 2021.

[15] PMDA. Conformance Guide on Electronic Study Data Submissions. 2015. http://www.pmda.go.jp/. Accessed 18 Jan 2021.

[16] Emilsson L, Lindahl B, Köster M, Lambe M, Ludvigsson JF (2015). Review of 103 Swedish Healthcare Quality Registries. J Int Med.

[17] Willis DG, Sullivan-Bolyai S, Knafl K, Cohen MZ (2016). Distinguishing features and similarities between descriptive phenomenological and qualitative description research. Western Journal of Nursing Research..

[18] Lincoln Y, Guba EG (1985). Naturalistic inquiry.

[19] DiCicco-Bloom B, Crabtree BF (2006). The qualitative research interview. Med Educ.

[20] Hennink MM, Kaiser BN, Marconi VC (2017). Code saturation versus meaning saturation: how many interviews are enough?. Qual Health Res.

[21] Harris PA, Taylor R, Thielke R, Payne J, Gonzalez N, Conde JG (2009). Research electronic data capture (REDCap)-a metadata-driven methodology and workflow process for providing translational research informatics support. J Biomed Inform.

[22] Harris PA, Taylor R, Minor BL, Elliott V, Fernandez M, O’Neal L (2019). The REDCap consortium: building an international community of software platform partners. J Biomed Inform.

[23] Tong A, Sainsbury P, Craig J. Consolidated criteria for reporting qualitative research (COREQ): a 32-item checklist for interviews and focus groups. Int J Qual Health Care. 2007;19(6):349-57. 10.1093/intqhc/mzm042. 10.1093/intqhc/mzm042 17872937

[24] Elo S, Kyngäs H (2008). The qualitative content analysis process. J Adv Nurs.

[25] Elliott D, Husbands S, Hamdy FC, Holmberg L, Donovan JL (2017). Understanding and improving recruitment to randomised controlled trials: qualitative research approaches. Eur Urol.

[26] Carlisle B, Kimmelman J, Ramsay T, MacKinnon N (2015). Unsuccessful trial accrual and human subjects protections: an empirical analysis of recently closed trials. Clin Trials.

[27] van den Bogert CA, Souverein PC, Brekelmans CTM, Janssen SWJ, Koëter GH, Leufkens HGM, Bouter LM (2017). Recruitment failure and futility were the most common reasons for discontinuation of clinical drug trials. Results of a nationwide inception cohort study in the Netherlands. J Clin Epidemiol.

[28] Campbell MK, Snowdon C, Francis D, Elbourne D, McDonald AM, Knight R (2007). Recruitment to randomised trials: strategies for trial enrollment and participation study. The STEPS study. Health Technol Assess (Winchester, England).

[29] Aamdal S. Cross-Nordic participation in clinical trials. Towards personalised medicine in Nordic clinical trials cooperation: NTA 2.0. Presentation in the 4th Nordic RWE conference. 2020. https://www.ascro.se/wp-content/uploads/2021/04/8_Aamdal_RecruitingPatientsfromothernordiccountries.pdf.

[30] Dombeck CB, Hinkley T, Fordyce CB, Blanchard K, Roe MT, Corneli A (2020). Continued investigator engagement: reasons principal investigators conduct multiple FDA-regulated drug trials. Contemp Clin Trials Commun.

[31] Cheng SK, Dietrich MS, Dilts DM (2010). A sense of urgency: evaluating the link between clinical trial development time and the accrual performance of cancer therapy evaluation program (NCI-CTEP) sponsored studies. Clin Cancer Res.

[32] Van Velthoven MH, Mastellos N, Majeed A, O’Donoghue J, Car J (2016). Feasibility of extracting data from electronic medical records for research: an international comparative study 90. BMC Med Inform Dec Mak.

[33] Lamberti MJ, Smith Z, Henry R, Howe D, Goodwin M, Williams A, Getz K (2020). Benchmarking patient recruitment and retention practices. Ther Innov Regul Sci.

[34] Safran C, Bloomrosen M, Hammond WE, Labkoff S, Markel-Fox S, Tang PC, et al. Toward a national framework for the secondary use of health data: an American Medical Informatics Association White Paper. J Am Med Inform Assoc [Internet]. 2007 Jan [cited 2020 Dec 12];14(1):1–9. 10.1197/jamia.M2273 PMC2329823 17077452

[35] Bahr A, Schlünder I. Code of practice on secondary use of medical data in European scientific research projects. Int Data Priv Law [Internet]. 2015 [cited 2020 Dec 15];5(4):279–291. Available from: 10.1093/idpl/ipv018

[36] Könberg P. The Future Nordic Co-operation on Health. Copenhagen: Nordisk Ministerråd; 2014. p. 32. 10.6027/ANP2014-731.

[37] Nordic NECT - Home [Internet]. [cited 2020 Sep 10]. Available from: https://nordicnect.org/

[38] Clement C, Selman LE, Kehoe PG, Howden B, Lane JA, Horwood J. Challenges to and facilitators of recruitment to an Alzheimer’s disease clinical trial: a qualitative interview study. J Alzheimer’s Dis. IOS Press. 2019;(4):69, 1067–1075. 10.3233/JAD-190146. 10.3233/JAD-190146 PMC6598018 31156168

[39] McCall SJ et al. Reporting transparency and completeness in trials: Paper 4 - reporting of randomised controlled trials conducted using routinely collected electronic records - room for improvement. J Clin Epidemiol. 2021;S0895-4356(21)00291-2. 10.1016/j.jclinepi.2021.09.011. 10.1016/j.jclinepi.2021.09.011 PMC8982641 34525409

